# Neuroserpin: A Potential Neuroprotective Agent in Mild Neonatal Hypoxic–Ischaemic Encephalopathy

**DOI:** 10.3390/cells14231840

**Published:** 2025-11-21

**Authors:** Eri Kawashita, Yumi Fukuzaki, Jan Fischer, Lei Shi, Yumei Liao, Lancelot Jamie Millar, Peiyun Zhong, Anna Hoerder-Suabedissen, Luana Campos Soares, Zoltán Molnár

**Affiliations:** 1 Department of Physiology, Anatomy and Genetics, University of Oxford, Sherrington Building, Sherrington Rd, Oxford OX1 3PT, UK; yumi.fukuzaki@ucsf.edu (Y.F.); t_shilei@jnu.edu.cn (L.S.); anna.hoerder-suabedissen@trinity.ox.ac.uk (A.H.-S.); luana.campossoares@dpag.ox.ac.uk (L.C.S.); 2 Laboratory of Pathological Biochemistry, Kyoto Pharmaceutical University, Misasagi Nakauchi-cho, Yamashina-ku, Kyoto 607-8414, Japan; 3 Department of Neurology, University of California, San Francisco, Sandler Neuroscience Building, 675 Nelson Rising Lane, San Francisco, CA 94158, USA; 4 JNU-HKUST Joint Laboratory for Neuroscience and Innovative Drug Research, College of Pharmacy, Jinan University, Guangzhou 510632, China

**Keywords:** neonatal hypoxic–ischaemic encephalopathy, Rice–Vannucci model, neuroserpin, neuroprotection

## Abstract

Neonatal hypoxic–ischaemic encephalopathy (HIE) remains a leading cause of infant morbidity and mortality worldwide, with therapeutic hypothermia being the only clinically approved treatment. This study investigates the cortical expression pattern of neuroserpin during postnatal brain development and evaluates its neuroprotective potential in hypoxia–ischaemia (HI)-induced brain damage using a modified Rice–Vannucci model. Experiments were conducted in both male and female neuroserpin knockout (KO) mice and through administration of exogenous neuroserpin into the brain. Between postnatal day 4 to 14 (P4–P14), neuroserpin-immunoreactive cell density peaked at P8–P10 in cortical layers 5 and 6b, with a gradual increase in layers 2/3 and minimal changes in layers 4 and 6a. Despite comparable levels of ischaemic brain damage between the KO and wild-type (WT) mice, exogenous neuroserpin administration suppressed the HI-induced oxidative stress. Additionally, it reduced microglial activation and reactive astrogliosis in the cortex in mild HIE, mitigating cortical thinning and preserving neuronal distribution. These findings suggest that endogenous neuroserpin alone is insufficient for neuroprotection against HI-induced damage, but exogenous neuroserpin shows promise as a pharmacological intervention for mild neonatal HIE.

## 1. Introduction

Neonatal hypoxic–ischaemic encephalopathy (HIE) is a neurological condition characterised by cellular and structural brain damage caused by insufficient oxygen delivery, leading to focal or global hypoxia and ischaemia [1]. Annually, over 1 million infants worldwide are affected by HIE, with high mortality rates and lifelong neurological sequelae, including movement disorders, cognitive and language deficits, and seizures [2,3]. The extent of brain injury and its outcomes in neonatal HIE depends on factors such as the degree and duration of hypoxia/ischaemia (HI) and the stage of brain maturation at the time of the insult. Clinical tools such as Sarnat staging, modified Sarnat staging, and Thompson score are used to classify neonatal HIE severity (mild, moderate, or severe) and to determine eligibility for therapeutic hypothermia, which is currently approved only for infants with moderate to severe HIE [4,5]. This treatment involves cooling the body or head to 33.5 ± 0.5 °C or 34.5 ± 0.5 °C to slow metabolism and reduce cellular energy demands, thereby significantly improving clinical outcomes [6,7,8,9,10]. However, despite its proven benefits, therapeutic hypothermia alone is insufficient to fully prevent brain injury, with approximately half of treated infants still experiencing death or long-term neurological disabilities [11,12]. Moreover, recent follow-up studies have revealed that even mild HIE can lead to subtle yet persistent neurodevelopmental impairments [13,14,15,16], whose pathophysiology is unclear. The accumulated evidence underscores the urgent need to develop adjunctive or alternative pharmacotherapeutic strategies that provide neuroprotection and improve neurodevelopmental outcomes in neonatal HIE.

The primary pathogenic factors underlying brain damage in neonatal HIE include excitotoxicity, oxidative stress, inflammation, mitochondrial dysfunction, and endoplasmic reticulum (ER) stress, which collectively lead to cell death through apoptosis, necrosis, and/or ferroptosis [9,17]. During the primary phase of energy failure caused by hypoxia and ischaemia (HI), ATP depletion, cytosolic calcium accumulation, neuronal depolarisation, and excessive glutamate release trigger excitotoxicity. Although there is a brief recovery period during reperfusion (latent phase), persistent excitotoxicity, inflammation, oxidative stress and mitochondrial dysfunction drive cell death (apoptosis and/or necrosis) in the secondary and tertiary phases. Activated microglia, astrocytes and neutrophils exacerbate brain injury by releasing inflammatory cytokines, chemokines, free radicals, and proteases, which increase blood–brain barrier (BBB) permeability and contribute to neurotoxicity [18]. Importantly, these pathogenic factors can also disrupt key processes of brain maturation, such as synaptogenesis, myelination, and astrocyte proliferation during postnatal development [19].

Several pharmacotherapeutic candidates targeting inflammation and oxidative stress, including erythropoietin and melatonin, have shown promise in clinical and anatomical studies of neonatal HIE [9,10,17]. However, given the multifactorial nature of HIE pathology, identifying complementary or alternative targets for neuroprotection is crucial. Neuroserpin, an endogenous inhibitor of tissue plasminogen activator (tPA), is highly conserved across species [20,21], and is expressed in neonatal and adult brains [22,23]. Neuroserpin’s neuroprotective properties have been demonstrated in adult rodent models of middle cerebral artery occlusion (MCAO) [24,25,26] and in vitro in oxygen-glucose deprivation and reoxygenation (OGD/R) models [27,28,29]. These studies suggest that neuroserpin mitigates NMDA receptor-mediated excitotoxicity, oxidative stress, inflammation, and ER stress, thereby reducing cell death and brain injury in adult stroke. Given the overlap in pathogenic mechanisms between adult stroke and neonatal HIE, neuroserpin holds significant potential as a candidate for the treatment of neonatal HIE [9,30]. Recent evidence highlights neuroserpin’s potential in HIE models. tPA knockout was shown to attenuate BBB permeability in a neonatal HIE model [31], and neuroserpin administration reduced hippocampal neuronal death in a similar model [32]. However, the effects of neuroserpin on cortical damage and its underlying mechanisms remain unclear. Given that mild HIE is increasingly recognized as a clinically relevant yet understudied condition [15,16], and that the efficacy of therapeutic hypothermia in this population remains uncertain, this study aimed to elucidate the pathophysiology of mild HIE and the therapeutic potential of neuroserpin. To reproduce this condition, we employed a milder version of the Rice–Vannucci model using 10% oxygen exposure for 40 min, reflecting emerging concerns that mild HIE, in the absence of gross infarction, can induce subtle but significant neurobiological alterations that may underlie subsequent neurodevelopmental impairments. Using this model, we examined the effects of both neuroserpin knockout and exogenous neuroserpin administration on cortical injury following HI.

## 2. Materials and Methods

### 2.1. Mouse Model of Neonatal HIE

To model mild HIE, we used two variants of the Rice–Vannucci method, with or without ligation of the external carotid artery (ECA). The method without ECA ligation was used to investigate the impact of neuroserpin deficiency on brain damage, whereas the method with ECA ligation was used to assess the effect of HI on neuroserpin expression and to evaluate the protective effects of exogenous neuroserpin (Supplementary Table S1).

To investigate the impact of neuroserpin deficiency on brain damage in HIE, a modified Rice–Vannucci model [33] was used with neuroserpin knockout (KO) mice (Serpini1^tm1Dpw^ (B6.129-Serpini1tm1Dpw/J), stock number 019121, The Jackson Laboratory, Bar Harbor, ME, USA) and wild-type (WT) mice. Neuroserpin KO mice were generated on a mixed 129/Sv and C57BL/6JBom background via neo cassette insertion in the second exon [34]. At postnatal day 8 (P8), when mouse brain development is comparable to the late third trimester or early neonatal period in humans [19], unilateral common carotid artery (CCA) ligation, followed by hypoxia (10% oxygen in a nitrogen gas mixture), was performed. Mice were anaesthetised with vaporised isoflurane (3.5% induction and 2% maintenance), and the left CCA was ligated with a 6-0 vicryl suture (W9981, Ethicon, Somerville, NJ, USA). After recovery, pups were returned to their cages for at least 1 h before exposure to hypoxia at 37 °C for 40 min. At P10, the pups were transcardially perfused with phosphate-buffered saline (PBS, 0.1 M, pH 7.4). These animal experiments were approved by a local ethical review committee of University of Oxford and conducted in accordance with personal and project licenses under the UK Animals (Scientific Procedures) Act (1986) (approval codes: P1E785A22 and PP0546018).

To evaluate the protective effects of exogenous neuroserpin, another modified Rice–Vannucci model was employed, in which both the CCA and ECA were permanently ligated [35]. This model was selected because it produces a more consistent and reproducible ischemic injury, thereby reducing inter-animal variability and allowing for more reliable assessment of neuroserpin’s effects [35]. C57BL/6J mice (Oriental Bio Service Inc., Kyoto, Japan) at P8 were anaesthetised with vaporised isoflurane (3% induction and 2% maintenance), and underwent left CCA and ECA ligation with 6-0 silk sutures (DEWB0603, Akiyama-seisakusyo Co. Ltd., Tokyo, Japan). Bupivacaine was applied topically to the surgical site to provide local analgesia. The mice were allowed to rest for at least 1 h, and then exposed to hypoxic gas (10% oxygen in a nitrogen gas mixture) at 37 °C for 40 min. Thirty minutes after hypoxia, the pups were injected into the lateral ventricle (coordinates: 1.1 mm laterally and 3.0 mm caudally from the lambda and −2.3 mm vertically from the cranial surface) with either vehicle (artificial cerebrospinal fluid, ACSF) (Tocris Bioscience, Minneapolis, MN, USA) or 100 ng of neuroserpin (PeproTech, Rocky Hill, NJ, USA, dissolved in ACSF at a concentration of 50 μg/mL, total volume of 2 μL) using a pulled glass capillary needle attached to a Hamilton syringe (701RN, Merck Life Science UK Ltd., Dorest, UK) under anesthesia (3% induction and 2% maintenance). After injection, the injector was held at the site for 1 min to prevent reflux [36]. The dose of 100 ng neuroserpin was selected based on our previous study in adult mice (weighing 20–25 g) in a stroke model, in which intracerebroventricular administration of both 100 ng (4–5 ng/g) and 500 ng (20–25 ng/g) exerted significant neuroprotective effects [26]. In the present study, P8 mice weighed approximately 4.2 ± 0.15 g, corresponding to a dose of ~24 ng/g. In the sham group, the suture was passed under the CCA and ECA without ligation. The mice were allowed to rest for at least 1 h, placed in an ambient air (21% oxygen) chamber maintained at 37 °C for 40 min, and then received an equivalent volume of vehicle. The pups were transcardially perfused with saline and/or 4% PFA in PBS either 24 h or 4 days later. Although this modified Vannucci method generally resulted in mild HIE, occasional severe cortical injury was observed. The severity was categorized based on gross cortical shape: animals exhibiting an obvious cortical depression were classified as severe, whereas those without were classified as mild. For the P2 HI model, C57BL/6J mice at P2 underwent left CCA and ECA ligation, were allowed to recover for at least 1 h, and were then exposed to hypoxic gas (10% oxygen in nitrogen) for 40 min. Recombinant neuroserpin (50 ng in 1 μL) or ACSF was administered intracerebroventricularly (0.9 mm lateral, 1.55 mm caudal to lambda, −1.2 mm from the skull surface) 30 min after the HI procedures. P2 mice weighed approximately 2.2 ± 0.05 g, corresponding to a dose of ~23 ng/g. These animal experiments were approved by the institutional animal care and use committee of Kyoto Pharmaceutical University and were performed in accordance with the institutional guidelines (approval code: A22-062-01).

No adverse events were observed during the procedures. Animals were monitored daily for any sign of distress and declining health, including weight loss and abnormal posture. A humane endpoint was defined as weight loss of 15% or more loss of body weight or signs of severe distress. Each dam and her litter were housed in a separate cage on paper chip bedding, under specific pathogen-free conditions, with a 12 h light/dark cycle and ad libitum access to food and water.

### 2.2. Cell Culture

Primary cortical neurons were prepared and cultured as described previously [37]. Briefly, pregnant Sprague Dawley rats at E18 were sacrificed by cervical dislocation. The cortices from the embryos were dissected out in ice-cold HBSS buffer (Life Technologies, Waltham, MA, USA) supplemented with 0.06% D-glucose, 1 mM sodium pyruvate, 10 mM HEPES buffer and 1% penicillin-streptomycin, and digested in 0.05% trypsin at 37 °C for 15 min with a shake every 2 min. Primary cortical neurons were dissociated by pipetting 15–20 times in Neurobasal medium (21103049, Gibco, Carlsbad, NM, USA) supplemented with 2% B27, 2 mM L-glutamine, 10 mM D-glucose and 1% penicillin-streptomycin (Life Technologies), and plated on poly-L-lysine (Sigma-Aldrich, St. Louis, MO, USA, 0.1 mg/mL)-coated culture dishes (8 × 10^5^ cells/35 mm dish). After 7 days in vitro (DIV), with half-medium changes every 3 days, neurons were used for the following experiments. The animal experiments were approved by the animal care and use committee of the animal facility at Jinan University and were conducted in accordance with the institutional guidelines (approval code: IACUC-20210330-15).

To investigate the effect of HI insult on neuroserpin expression in cultured neurons, an oxygen-glucose deprivation reperfusion model was used. Briefly, the cortical neurons at 7 DIV were cultured with glucose-free DMEM medium in a Modular Incubator Chamber (Billups-Rothenberg, Del Mar, CA, USA) for 4 h with hypoxic environmental conditions (95% N_2_ and 5% CO_2_). Then, cortical neurons were refreshed with the standard culture medium under normoxic conditions for reperfusion.

### 2.3. Experimental Design

The sample sizes were determined based on feasibility and ethical considerations regarding the use of animals. Animals and cell culture samples were randomly assigned to control and treatment groups to minimise bias, although no formal method of randomisation was used. Group allocation was performed by EK, JF, LS, and YL. To ensure blinding, each sample was labelled with an ID unrelated to the group name. During data analysis, the investigators were unaware of the group assignments corresponding to each ID. To minimise potential confounders, the order of treatments and measurements was randomised. Both male and female animals were used, but no distinction was made between sexes in the analysis. Animals were randomly assigned to each experimental group, and no stratification by sex was performed. All animal study protocols, including the research question, design and statistical plan, were prepared and approved by the institutional animal ethics committee prior to the start of the study, although it was not registered in a public database.

### 2.4. Immunohistochemistry

For developmental neuroserpin immunostaining, brains from P4–P14 and adult mice were perfused with PBS followed by 4% PFA in PBS and fixed in 4% PFA for 48 h. Brains were embedded in 5% agarose, and then sectioned coronally (80 µm thickness) using a vibrating microtome (VT1000S, Leica, Wetzlar, Germany). After washing with PBS, the floating sections were incubated in 2% donkey serum (D9663, Sigma-Aldrich, St. Louis, MO, USA) in PBS containing 0.2% Triton X-100 as a blocking solution. The sections were then incubated with rabbit anti-neuroserpin polyclonal antibody (ab33077, Abcam, Cambridge, UK; diluted 1:1000 with blocking solution) and goat anti-connective tissue growth factor (CTGF) polyclonal antibody (sc-14939, Santa Cruz Biotechnology, Santa Cruz, CA, USA; diluted 1:1000 with blocking solution) at 4 °C overnight. Subsequently, the sections were washed with PBS, and then incubated with the following secondary antibodies for 2 h at room temperature (RT): Alexa Fluor 488-conjugated donkey anti-rabbit IgG (A21206, Thermo Fisher Scientific, Waltham, MA, USA; diluted 1:500 with blocking solution) and Alexa Fluor 568-conjugated donkey anti-goat IgG (A11057, Thermo Fisher Scientific; diluted 1:500 with blocking solution). Sections were mounted on glass slides and imaged using a confocal laser microscope (LSM 710, Carl Zeiss, Oberkochen, Germany). Neuroserpin-immunoreactive cells were quantified in a blinded manner using ImageJ software (version 1.52) with the Analyse Particles plugin (NIH, Bethesda, MD, USA). Heatmaps were generated by averaging cortical images and overlaying them using ImageJ with the bUnwarpJ plugin (version 2.6.8).

For HIE brain immunostaining, brains were perfused with saline and 4% PFA in PBS, fixed in 4% PFA for 24 h, then cryoprotected in 30% sucrose for 48 h. After washing with PBS, brains were snap-frozen in 2-methylbutan cooled with dry ice, and sectioned coronally (20 µm thickness) using a cryostat (CM1860, Leica). Slide-mounted sections were blocked using mouse-on-mouse blocking kit (Vector Laboratories, Newark, CA, USA) and incubated with blocking solution (0.3% Triton X-100 and 20% normal goat serum (Vector Laboratories) in PBS). The sections were then incubated with the following primary antibodies in 5% normal goat serum in PBS containing 0.1% Triton X-100 for 48 h at 4 °C: mouse anti-NeuN monoclonal antibody (MAB377, Chemicon International Inc., Temecula, CA, USA; diluted 1:1000), rat anti-CD68 antibody (ab53444, Abcam; diluted 1:200), rabbit anti-neuroserpin antibody (ab33077, Abcam; diluted 1:200), chicken anti-glial fibrillary acidic protein (GFAP) antibody (ab4674, Abcam; diluted 1:200), and rat anti-myelin basic protein (MBP) antibody (ab7349, Abcam; diluted 1:200). Sections were washed with PBS, and then incubated with Alexa Fluor 488-conjugated goat anti-mouse IgG (A11001, Thermo Fisher Scientific; diluted 1:250), Alexa Fluor 568-conjugated goat anti-rat IgG (A11077, Thermo Fisher Scientific; diluted 1:250), Alexa Fluor 647-conjugated goat anti-rabbit IgG (A21245, Thermo Fisher Scientific; diluted 1:250), and Alexa Fluor 647-conjugated goat anti-chicken IgY (A21449, Thermo Fisher Scientific; diluted 1:250) for 2 h at RT. After nuclear staining with 4′,6-diamidino-2-phenylindole (DAPI, D523, Dojin Kagaku, Tokyo, Japan; diluted 1:800 in PBS), the sections were mounted on glass slides and imaged using an all-in-one fluorescence microscope (BZ-X800 or BZ-X1000, Keyence, Osaka, Japan). Animals that showed no detectable brain injury, as assessed by the absence of CD68 immunoreactivity in any brain region, were considered unsuccessful in model generation and were excluded from subsequent analyses. This criterion was predefined. There was no notable difference in model success rate between the control and treatment groups (2 out of 9 animals and 1 out of 9 animals, respectively). CD68- or GFAP-immunoreactive areas, the number of NeuN-immunoreactive cells, cortical thickness, the mean intensity of MBP immunoreactivity, and myelinated cortex areas were measured in a blinded manner using ImageJ software (version 1.54; NIH). The number of neuroserpin-immunoreactive cells was manually counted in a blinded manner.

### 2.5. Immunoblotting

After perfusion with PBS, mouse brain hemispheres were homogenised and sonicated in lysis buffer containing 50 mM Tris-HCl (pH 7.4), 150 mM NaCl, 1% Nonidet P-40, 0.5% sodium deoxycholate, and 0.1% sodium dodecyl sulfate, supplemented with a complete protease inhibitor cocktail tablet (Roche Diagnostics, Mannheim, Germany) and phosphatase inhibitor cocktail solution (Wako Pure Chemical Industries, Osaka, Japan). The supernatant was collected after centrifugation at 17,800× *g* for 15 min at 4 °C. Protein concentrations were determined using a BCA protein assay kit (Pierce, Rockford, IL, USA). Equal amounts of protein from each lysate were subjected to gel electrophoresis (SDS-polyacrylamide) on 5–20% polyacrylamide gels (e-PAGEL HR, Atto, Tokyo, Japan), and transferred onto nitrocellulose membranes. Membranes were blocked with 3% skim milk in Tris-buffered saline containing 0.05% Tween-20 (TBS-T) and incubated with mouse anti-hexanoyl-lysine (HEL) antibody (MHL-021P, Japan Institute for the Control of Ageing, Shizuoka, Japan; diluted 1:500 with Can Get Signal Solution 1 (Toyobo, Osaka, Japan)) and rabbit anti-neuroserpin antibody (ab33077, Abcam; diluted 1:500 with blocking solution) at 4 °C overnight. Following washes with TBS-T, the membranes were incubated with horseradish peroxidase (HRP)-conjugated anti-mouse IgG (sc-516102, Santa Cruz Biotechnology; diluted 1:2500 with Can Get Signal Solution 2 (Toyobo)) and HRP-conjugated anti-rabbit IgG (111-035-144, Jackson ImmunoResearch Laboratories, West Grove, PA, USA; diluted 1:2500 with blocking solution) for 1 hour at RT. After washing, immunoreactive bands were detected with Chemi-Lumi One Super (Nacalai Tesque) using a LAS-3000 mini-image analysis system (Fujifilm, Tokyo, Japan). The membranes were then immersed in a stripping solution (193-16375, Wako Pure Chemical) for 15 min at RT, washed with TBS-T, and used for glyceraldehyde-3-phosphate dehydrogenase (GAPDH) detection with mouse anti-GAPDH antibody (016-25523, Wako Pure Chemical Industries; diluted 1:2000 with blocking solution) and HRP-conjugated anti-mouse IgG (Santa Cruz Biotechnology). Band intensities were quantified using ImageJ software (NIH). The same sample was included in each Western blot analysis as a loading control, and band intensities were normalised. The band intensities of neuroserpin protein were divided by those of GAPDH protein, and the relative expression to the control group was calculated.

For the cultured neurons, immunoblotting of neuroserpin was conducted after oxygen-glucose deprivation reperfusion with different times (0, 0.25, 0.5, 1, 2, 4, 6, 8, 12, 24 h). Cortical neurons were lysed in RIPA buffer supplemented with protease inhibitor cocktail, and the supernatant was collected after centrifugation with 15,294× *g* for 15 min at 4 °C. The Pierce BCA protein assay kit (Thermo Scientific, Waltham, MA, USA) was used to determine the protein quantitation. Equal protein amounts were separated by 10% SDS-PAGE gel and transferred to PVDF membranes. After blocking with 5% skim milk in TBS-T and washing three times with TBS-T, the blocked membranes were incubated with antibodies against neuroserpin (mouse, 1:1000; sc-48360, Santa Cruz Biotechnology) and GAPDH (rabbit, 1:2000; 10494-1-AP, Proteintech, Rosemont, IL, USA) overnight at 4 °C. After washing three times with TBS-T, the membranes were incubated with goat anti-mouse IgG H&L (HRP) (1:2000; 7076S, Cell Signaling Technology, Danvers, MA, USA) and goat anti-rabbit IgG H&L (HRP) (1:4000; 7074S, Cell Signaling Technology) for 1 hour at RT. After washing, immunoreactive bands were visualized using enhanced chemiluminescence (GE Healthcare, Chicago, IL, USA) and imaged by Amersham Imager 600 (GE Healthcare). For quantification of protein levels, the band gray value was analysed using Quantity One software (version 4.6.2.70, Bio-Rad, Hercules, CA, USA), and the value of neuroserpin protein was divided by the internal reference protein to obtain the relative expression. The relative expression in the experimental group was normalized to that in the control group.

### 2.6. Statistical Analyses

All data are presented as mean ± standard error of the mean (SEM). No data points were excluded during statistical analysis. Statistical differences between group means were analysed using appropriate tests based on experimental design, using GraphPad Prism 10 software. The normality of data distribution was assessed using the Shapiro–Wilk test or Q-Q plots. Homogeneity of variances was evaluated using the F-test for t-tests, and the Brown–Forsythe test or residual plots for one-way and Two-way analysis of variance (ANOVA). These included Student’s t-test for comparisons between two groups, one-way ANOVA, followed by Tukey’s or Bonferroni’s multiple comparison post hoc tests for multi-group comparisons. Two-way ANOVA, followed by Tukey’s post hoc test for factors with two independent variables, and Fisher’s exact test for categorical data analysis were used. When assumptions for normality and homogeneity were not met, non-parametric alternative tests were applied: the Mann–Whitney U test for comparisons between two groups and the Kruskal–Wallis test followed by Dunn’s multiple comparisons test for multi-group comparisons. Detailed *p*-values for ANOVA and Kruskal–Wallis tests are provided in Supplementary Table S2. A *p*-value of less than 0.05 was considered statistically significant.

## 3. Results

### 3.1. Transition of Neuroserpin Expression in the Cortex During Mouse Postnatal Development

Our previous study in human brain development demonstrated that the subplate and deep cortical plate neurons are the primary sources of neuroserpin from the 25th gestational week until the first postnatal month, and that neuroserpin expression expands towards the upper cortical layers after the 3rd postnatal month [23]. Furthermore, we showed that neuroserpin is highly expressed in the subplate neurons of the mouse brain at P8, which is approximately equivalent to the human full-term [22]. However, the detailed spatiotemporal patterns of neuroserpin during neonatal mouse brain development remain unknown. We therefore examined the expression pattern of endogenous neuroserpin during the crucial time span of P4–P14.

**Figure 1:**
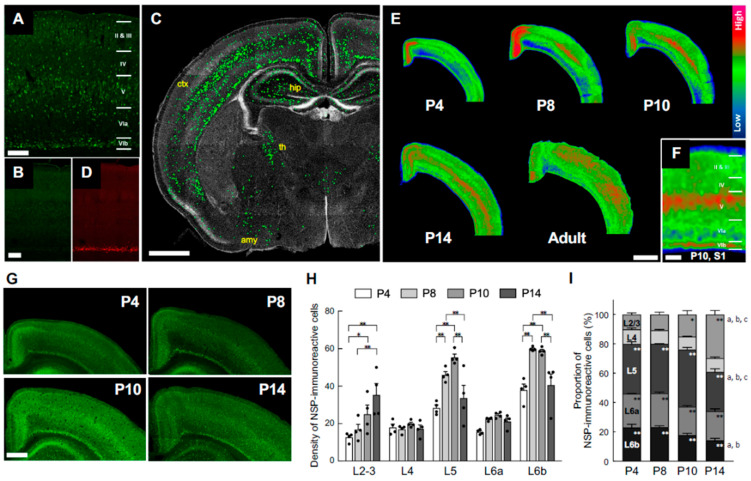
Cortical neuroserpin expression pattern during postnatal brain maturation. (**A**,**B**) Representative images of neuroserpin immunoreactivity in S1 of wild-type (WT) mice (**A**) and neuroserpin knockout (KO) mice (**B**) at P10. The scale bars indicate 200 µm; the bar in (**B**) also applies to (**D**). (**C**) Distribution of neuroserpin-immunoreactive cells as seen in the coronal section of a representative P10 mouse brain (ctx: cerebral cortex; hip: hippocampus; th: thalamus; amy: amygdala). The immunoreactivity of the individual cells was enhanced by ImageJ to be visible at the given magnification. The scale bar indicates 1 mm. (**D**) Representative image of connective tissue growth factor (CTGF) immunoreactivity in S1 of neuroserpin KO mice. (**E**) Representative heatmaps of neuroserpin immunoreactivity in the parietal cortex at various postnatal ages (P4, 8, 10, 14, and adult). The scale bar indicates 1 mm. (**F**) Magnified heatmap of neuroserpin immunoreactivity in S1 at P10. Layers V and VI have the strongest immunoreactivity. The scale bar indicates 200 µm. (**G**) Representative images of neuroserpin immunoreactivity in the cortex at P4, 8, 10, and 14. The scale bar in P10 indicates 500 µm and applies to all panels. (**H**) Density of neuroserpin-immunoreactive cells in each of the layers (*n* = 4 at each age group; total of 16 animals used). Values represent the mean ± standard error of the mean (SEM). Statistical significance was evaluated using a two-way analysis of variance (ANOVA) followed by a Tukey post hoc test. * *p* < 0.05, ** *p* < 0.01. (**I**) Relative proportion of total number of neuroserpin-immunoreactive cells in each layer (*n* = 4 at each age group; total of 16 animals used). Values represent the mean ± SEM. Statistical significance was evaluated using a two-way analysis of ANOVA followed by a Tukey post hoc test. * *p* < 0.05, ** *p* < 0.01 vs. L4 at each age. ^a^ *p* < 0.01 P4 vs. P14 in each layer. ^b^ *p* < 0.01 P8 vs. P14 in each layer. ^c^ *p* < 0.01 P10 vs. P14 in each layer.

Neuroserpin immunoreactivity was absent in brain sections from neuroserpin KO mice, confirming both the specificity of the antibody and the loss of neuroserpin expression in the KO mice (Figure 1A,B). At P10, a notable expression of neuroserpin was observed in the cerebral cortex, with additional presence in the hippocampus, amygdala, and lateral thalamus (Figure 1C). It is noteworthy that neuroserpin-immunoreactive cells were abundant in layer 6b (L6b) (Figure 1A), which exhibited selective expression of CTGF (Figure 1D), as well as in layer 5 (L5) (Figure 1A). In contrast, there was a relative scarcity of neuroserpin-immunoreactivity in layers 4 and 6a (Figure 1A). Neuroserpin immunoreactivity was detected in neurons in all cortical layers at the examined ages, ranging from P4, P14 to 6 months. However, the expression pattern of neuroserpin was not static during the first two postnatal weeks (Figure 1E–I). The heatmapping of neuroserpin immunoreactivity shows the dynamic alteration in the overall cortical expression patterns over time (Figure 1E). At P4, neuroserpin was observed to be enriched in L5 and L6b, notably in the retrosplenial cortex, the cortical area closest to the midline (Figure 1E,G). Subsequently, the density of neuroserpin-immunoreactive cells in L5 and L6b increased, particularly in the barrel field of the primary somatosensory cortex (S1), with peaks at P8–P10 (Figure 1E–H). In contrast to L5 and L6b, the density and the proportion of neuroserpin-immunoreactive cells in layers 2/3 (L2/3) increased continuously during the first two postnatal weeks, reaching the same extent as in L5 at P14 (Figure 1H,I). Notably, the number of neuroserpin-immunoreactive cells in L4 was significantly lower compared to other layers at P10 and P14 (Figure 1I). In the adult mouse cortex, neuroserpin expression was more uniformly distributed throughout the upper cortical layers, with minimal presence in L6b (Figure 1E). These results demonstrate that the transition of neuroserpin expression from the inner layers to all layers during cortical development is well conserved between humans and mice. Furthermore, the expression of neuroserpin in primary cortical neurons gradually increased from day in vitro (DIV) 1 to DIV14 (Supplementary Figure S1), suggesting that neuroserpin expression is associated with neuronal maturation.

### 3.2. Effects of Hypoxia–Ischaemia on Neuroserpin Expression in Primary Cultured Cortical Neurons and Mouse Cortex

**Figure 2:**
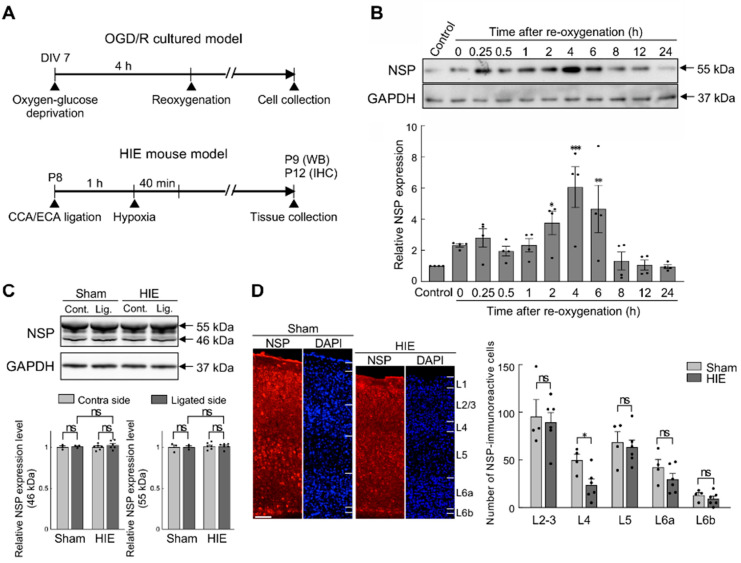
Neuroserpin expression in cultured cortical neurons exposed to oxygen-glucose deprivation and reoxygenation (OGD/R) and the mouse cortex of a neonatal HIE model. (**A**) Diagrams of the experimental procedures of the generation of the oxygen-glucose deprivation/reoxygenation (OGD/R) model and the neonatal HIE mouse model. In the in vitro HI model, primary cortical neurons at DIV 7 were exposed to OGD for 4 h, followed by reoxygenation. The cells were harvested at the specified time points. C57BL/6J mice at P8 were subjected to left CCA and ECA ligation, and then exposed to hypoxic gas (10% oxygen in a nitrogen gas mixture) for 40 min after a minimum of 1 h rest. Western blotting was performed on the hemicerebrums collected 24 h following the HI procedure. For immunohistochemical staining, brains were collected 4 days following the HI procedure. (**B**) A representative immunoblotting image of neuroserpin expression in cultured primary neurons at the indicated time points following OGD (upper panel). The band intensity was measured and normalized to that of GAPDH (lower panel) (*n* = 4 at each time point; total of 44 samples). Values represent the mean ± SEM. Statistical significance was evaluated using a one-way ANOVA followed by a Bonferroni multiple comparisons test. * *p* < 0.05, ** *p* < 0.01, *** *p* < 0.001. (**C**) Representative immunoblotting images of neuroserpin and GAPDH expression in the contra and ligated sides of the hemicerebrums of sham mice and HIE mice 24 h following the HI procedure (upper panel). The band intensity was measured using ImageJ software and normalised to that of GAPDH (lower panel) (sham mice: *n* = 3; HIE mice: *n* = 6; total of 9 animals used). Values represent the mean ± SEM. Statistical significance was evaluated using a one-way ANOVA followed by a Tukey post hoc test. ns: non-significant. (**D**) Representative immunostained images of neuroserpin in S1 of sham mice and mild HIE mice 4 days after the HI procedure (left panel). The scale bar indicates 100 µm. The number of neuroserpin-immunoractive cells in each cortical layer was counted in a blinded manner (left panel) (sham mice: *n* = 4; HIE mice: *n* = 6; group; total of 10 animals used). Values represent the mean ± SEM. Statistical significance was evaluated using a two-way ANOVA followed by a Tukey post hoc test. * *p* < 0.05, ns: non-significant.

As the protein level of neuroserpin is increased following HI insults in adult brains [24,38], we sought to investigate whether HI affects neuroserpin expression in cultured cortical neurons and the neonatal cortex in vivo. To this end, neuroserpin expression was analysed in an in vitro HI model and in neonatal mice subjected to the modified Rice–Vannucci procedure at P8 (Figure 2A). The expression of neuroserpin was increased in cultured cortical neurons after reoxygenation following oxygen-glucose deprivation, reaching a maximum level at 4 h and returning to control levels at 8 h (Figure 2B). In contrast, the expression levels of neuroserpin protein in the hemicerebrum were abundant and comparable between sham and HIE mice at 24 h after the HI procedure (Figure 2C). Additionally, there was no dramatic difference in the cortical expression pattern of neuroserpin between sham and mild HIE mice 4 days after the HI procedure, although the number of neuroserpin-immunoreactive cells in L4 tended to be lower in mild HIE mice compared to sham mice (Figure 2D). These results suggest that reoxygenation following HI might increase the cortical expression of neuroserpin, but this effect is not sustained over the long term.

### 3.3. No Difference in Neonatal Hypoxic–Ischaemic Brain Damage Between Neuroserpin KO Mice and WT Mice

**Figure 3:**
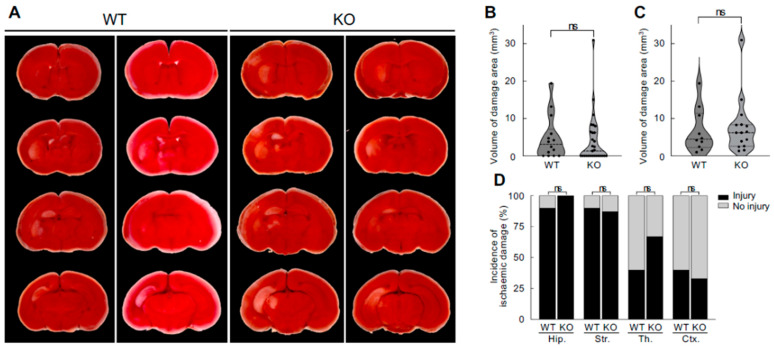
Effects of neuroserpin deficiency on HI-induced brain damage. (**A**) Representative 2,3,5-Triphenyltetrazolium chloride (TTC) staining images of the brains of P10 WT mice and neuroserpin KO mice following the HI procedure at P8. The red TTC staining marks viable tissue, while its absence (appears white) indicates the presence of brain damage. (**B**) Mean volume of damaged brain area in WT and neuroserpin KO mice was measured using ImageJ software (WT mice: *n* = 14; KO mice: *n* = 29; total of 43 animals used). Values represent the mean ± SEM. Statistical significance was evaluated using Mann–Whitney U test. ns: non-significant. (**C**) Mean volume of damaged brain area in WT and neuroserpin KO mice, with the exclusion of animals that did not sustain any brain injury (WT mice: *n* = 10; KO mice: *n* = 15; total of 25 animals used). Values represent the mean ± SEM. Statistical significance was evaluated using Mann–Whitney U test. (**D**) Incidence of HI-induced damage in each brain region (Hip: hippocampus; Str: striatum; Th: thalamus; and Ctx: cortex). Statistical significance between WT and KO groups in each brain region was evaluated using Fisher’s exact test.

To investigate whether endogenous neuroserpin plays a role in neuroprotection against neonatal HI brain damage, WT mice and neuroserpin KO mice were subjected to HI procedures according to the modified Rice–Vannucci model at P8, and the extent of HI brain damage was determined by 2,3,5-Triphenyltetrazolium chloride (TTC) staining at P10 (Figure 3). We expected more severe injury in KO mice even under the mild HI condition, but there was no significant difference in the volume of HI brain damage between WT mice and KO mice (WT: 4.79 ± 1.56 mm^3^; KO: 4.02 ± 1.22 mm^3^), given equivalent overall brain sizes (Figure 3A,B). We observed substantial inter-individual variability in the extent of injury, and some animals did not show any visible infarction on TTC staining, which likely reflects the limited reproducibility of ischemic injury in this model. Even when only the animals with metabolically inactive tissue were compared, the volume of the brain damage in the KO mice was comparable to that in WT mice (WT: 6.71 ± 1.87 mm^3^, KO: 7.78 ± 1.91 mm^3^) (Figure 3A,C). The analysis of the incidence of HI damage in each brain area showed that the hippocampus and striatum were more susceptible than the thalamus and cortex: the incidence was 90% in the hippocampus and striatum and 40% in the thalamus and cortex of WT mice (*p* = 0.03, Fisher’s exact test). Although the incidence of HI damage in the thalamus appeared to be higher in KO mice than in WT mice (WT: 40%, KO: 67%; *p* = 0.24), there was no significant difference between WT and KO mice in any of the brain regions examined (Figure 3D). During the early postnatal period, the cerebrovascular network and collateral circulation are still maturing, and the extent of collateral blood flow can vary between individuals, which likely contributes to variability in the severity and regional distribution of brain damage.

### 3.4. Neuroprotective Effect of Treatment with Neuroserpin on Brain Damage in Mild Neonatal HIE

**Figure 4:**
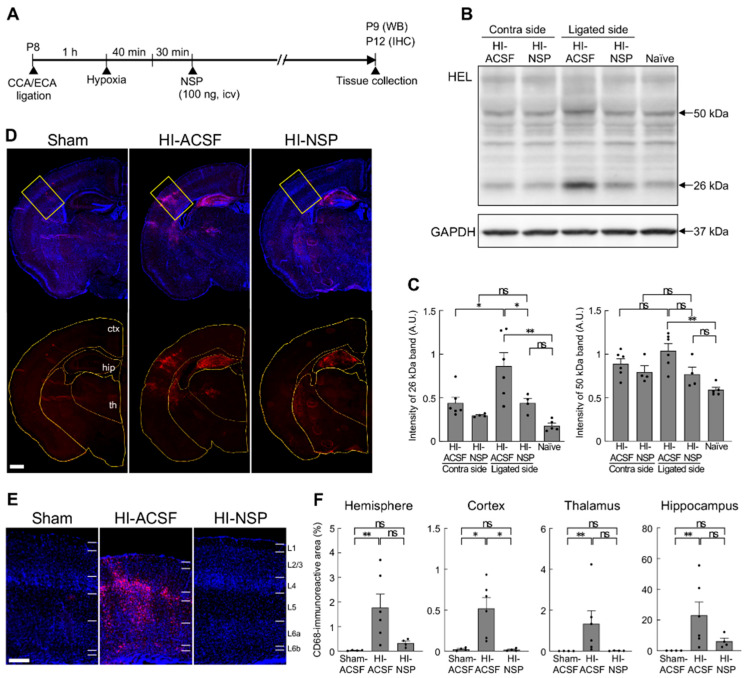
Effects of an intracerebroventricular injection of recombinant neuroserpin protein on HI-induced oxidative stress and microglial activation. (**A**) Diagrams of the experimental procedures for the generation of a neonatal HIE model and the administration of recombinant neuroserpin protein. C57BL/6J mice at P8 were subjected to left CCA and ECA ligation, allowed to rest for at least 1 h, and then exposed to hypoxic gas (10% oxygen in a nitrogen gas mixture) for 40 min. The pups were administered 100 ng of recombinant neuroserpin protein or ACSF intracerebroventricularly 30 min after HI procedures. Western blotting was performed on the hemicerebrums collected 24 h following the HI insult. For immunohistochemical analysis, brains were collected 4 days following the HI insult. HI-ACSF: mice that underwent HI insult and ACSF injection; HI-NSP: mice that underwent HI insult and neuroserpin injection. (**B**) Representative immunoblotting images of HEL and GAPDH expression in the contra and ligated sides of the hemicerebrums of HI-ACSF mice and HI-NSP mice 24 h following the HI procedure, and in the hemicerebrum of naïve mice. (**C**) The band intensity was measured using ImageJ software and normalised to that of GAPDH (HI-ACSF mice: *n* = 6; HI-NSP mice: *n* = 4; naïve mice: *n* = 5; total of 15 animals used). Values represent the mean ± SEM. Statistical significance was evaluated using a one-way ANOVA followed by a Tukey post hoc test. * *p* < 0.05, ** *p* < 0.01. ns: non-significant. (**D**) Representative images of CD68 immunostaining of brain sections including S1 from P12 sham mice, HI-ACSF mice and HI-NSP mice with mild HIE. The merged images of CD68 and DAPI staining (upper panel), and the images of CD68 immunostaining with a region of interest demarcating each brain region are presented (lower panel, ctx: cerebral cortex; th: thalamus; hip: hippocampus). The scale bar indicates 500 µm and applies to all panels. (**E**) Magnified immunostained images of CD68, indicated by the rectangle in Figure 4D, are presented. The scale bar indicates 200 µm. (**F**) The CD68-immunoreactive areas in the ligated side of the hemisphere, cortex, thalamus and hippocampus were measured using ImageJ software in a blinded manner (sham mice: *n* = 4; HI-ACSF mice: *n* = 6; HI-NSP mice: *n* = 4; total of 14 animals used). Values represent the mean ± SEM. Statistical significance was evaluated using a Kruskal–Wallis test followed by Dunn’s multiple comparisons test. * *p* < 0.05, ** *p* < 0.01. ns: non-significant.

To evaluate the effects of exogenous neuroserpin on brain damage in mild HIE, recombinant neuroserpin protein was injected into the lateral ventricle of WT mice 30 min after the HI procedure at P8, and the brains were harvested at P12. Since the considerable variability in terms of infarct presence and size was observed in the aforementioned HIE model, we used the modified Rice–Vannucci method with ligation of both the ipsilateral CCA and ECA. This modification reduces variability in infarct volume by limiting collateral blood flow in neonatal rodents [35]. Hypoxia was then induced by exposure to hypoxic gas for 40 min in this experiment (Figure 4A). As oxidative stress is one of the most critical factors contributing to brain damage in neonatal HIE [9,17], we first assessed the effect of neuroserpin injection on HI-induced oxidative stress by detecting the levels of 13-hydroperoxyoctadecanoic acid (13-HPODE)-modified protein (namely HEL), which indicates the level of lipid peroxidation (Figure 4B,C). As the administration of an inducer of oxidative stress (paraquat) resulted in increased levels of 26- and 50-kDa HEL in mouse brains [39], the intensity of these bands was measured in this study (Figure 4B,C). The intensity of the 26-kDa band was higher in the ligated side of the cerebral hemisphere of the HI-ACSF group (the mice that underwent HI insult and ACSF injection) than in the contralateral side, but there was no significant difference in the intensity of the 50-kDa band between the contralateral and ligated sides. The increased level of the 26-kDa HEL was significantly reduced by treatment with neuroserpin to the level observed in naïve mice, indicating that exogenous neuroserpin suppressed HI-induced oxidative stress. Furthermore, as neonatal HI induces the accumulation of activated microglia in injury regions [40,41], we investigated the effect of neuroserpin administration on microglial activation by detecting immunoreactivity for CD68, a marker of activated microglia with phagocytic activity (Figure 4D–F). The severity of HI brain damage was categorised as mild or severe based on cortical shape: brains with and without obvious cortical depression were classified as severe and mild damage, respectively. There was no significant difference in severity between the HI-ACSF group and the HI-NSP group (the mice that underwent HI insult and neuroserpin injection): the number of mice exhibiting severe and mild HI damage in the HI-ACSF group was 1 and 6, respectively, and the number of mice in the HI-NSP group was 4 and 4, respectively (*p* = 0.28, Fisher’s exact test). Marked CD68-immunoreactivity was observed in the cerebral hemisphere of HI mice, but not observed in the sham mice (Figure 4D–F). In the severe category, a single administration of neuroserpin was not sufficient to reduce the size of CD68-immunoreactive areas in either the cerebral hemisphere or any brain region of interest (cortex, hippocampus or thalamus) (Supplementary Figure S2). However, in the mild category, CD68-immunoreactive areas, particularly in the cortex, were significantly smaller in the HI-NSP group than in the HI-ACSF group (Figure 4D–F), indicating that neuroserpin suppressed microglial activation in mild HIE. It is noteworthy that the HI-ACSF group exhibited evident CD68-immunoreactivity in cortical L4 and it was clearly suppressed by treatment with neuroserpin. In contrast, no significant reduction in CD68-immunoreactivity was observed in the hippocampus following treatment with neuroserpin (Figure 4D,F).

**Figure 5:**
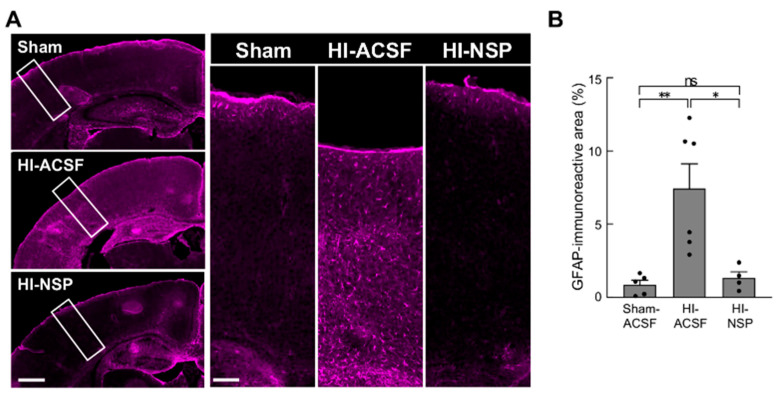
Effects of an intracerebroventricular injection of recombinant neuroserpin protein on HI-induced reactive astrogliosis. (**A**) Representative images of GFAP immunostaining in the brains of P12 sham mice, HI-ACSF mice and HI-NSP mice with mild HIE. The rectangle indicates the S1 region in high-magnification images, and the area was used to measure GFAP-immunoreactive area. C57BL/6J mice were subjected to HI procedures at P8, and then administered 100 ng of recombinant neuroserpin protein into the lateral ventricle of the brain 30 min after HI insults. The scale bars indicate 500 µm and 100 µm, in the left and right panels, respectively. (**B**) GFAP-immunoreactive were measured using ImageJ software in a blinded manner (sham mice: *n* = 5; HI-ACSF mice: *n* = 6; HI-NSP mice: *n* = 4; total of 15 animals used). Values represent the mean ± SEM. Statistical significance was evaluated using a one-way ANOVA followed by a Tukey post hoc test. * *p* < 0.05, ** *p* < 0.01. ns: non-significant.

In addition to microglial activation, we evaluated the effect of neuroserpin on reactive astrogliosis (Figure 5). At P12, GFAP-positive astrocytes were barely detectable in the cortex of sham mice (Figure 5A), although S100β immunostaining confirmed the presence of astrocytes (Supplementary Figure S3). In contrast, mild HIE induced a marked increase in GFAP-positive astrocytes throughout the cortical layers. Moreover, neuroserpin treatment significantly reduced the GFAP-immunoreactive area, suggesting that neuroserpin also suppressed reactive astrogliosis (Figure 5A,B).

**Figure 6:**
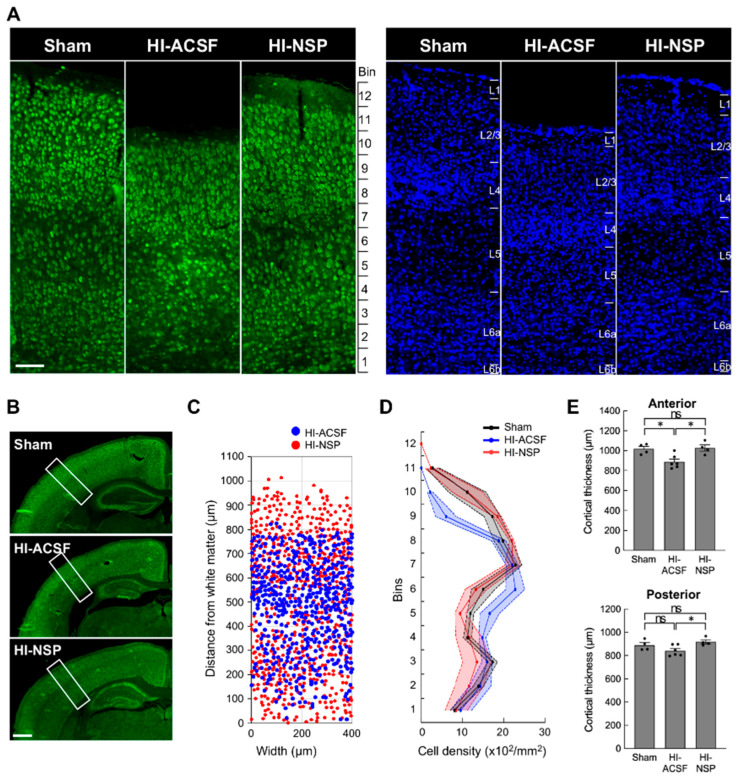
Effects of an intracerebroventricular injection of recombinant neuroserpin protein on HI-induced alteration in cortical distribution of neurons and reduction in cortical thickness. (**A**) Representative images of NeuN immunostaining (left panel) and DAPI staining (right panel) in the brains of P12 sham mice, HI-ACSF mice and HI-NSP mice with mild HIE. C57BL/6J mice were subjected to HI procedures at P8, and then administered 100 ng of recombinant neuroserpin protein into the lateral ventricle of the brain 30 min after HI insults. The scale bar indicates 100 µm and applies to all panels. (**B**) The rectangle indicates the S1 region in high-magnification images, and the area was used for cell counting. The scale bar indicates 500 µm. (**C**) Illustration of the distribution of NeuN-immunoreactive cells in the rectangular area shown in Figure 6B. (**D**) The density distribution of NeuN-immunoreactive cells in the rectangular area shown in Figure 6B (sham mice: *n* = 4; HI-ACSF mice: *n* = 6; HI-NSP mice: *n* = 4; total of 14 animals used). Values represent the mean ± SEM. (**E**) Cortical thickness of the anterior and posterior S1 is shown in the upper and lower panels, respectively. Cortical thickness was measured at three different locations in the rectangular area using ImageJ software in a blind manner, and mean value was calculated (sham mice: *n* = 4; HI-ACSF mice: *n* = 6; HI-NSP mice: *n* = 4; total of 14 animals used). Values represent the mean ± SEM. Statistical significance was evaluated a one-way ANOVA followed by a Tukey post hoc test. * *p* < 0.05, ns: non-significant.

Since neuroserpin exerted protective effects against glial activation in mild HIE, we further examined whether the laminar distribution of mature neurons was affected in mild HIE and whether the alteration was suppressed by treatment with neuroserpin. To this end, we analysed the density of NeuN-immunoreactive cells in 100 μm bins in S1 at P12, following HI at P8. The distribution of the NeuN-immunoreactive cells was altered after HI, but this alteration was prevented by neuroserpin treatment, maintaining levels similar to sham controls (Figure 6A–D). In addition, cortical thickness was reduced in the HI-ACSF group compared to the sham group, but the reduction was significantly attenuated by treatment with neuroserpin (Figure 6A,E). In the context of cortical development, laminar maturation occurs in deep layers (L5–6) by P2 in mice, and upper layers (L1–4) appear by P8 [42,43,44]. Therefore, these results suggest that neuroserpin exerts neuroprotective effects against HI-induced cortical damage and/or impaired laminar maturation in mild neonatal HIE. Notably, the laminar distribution of NeuN-immunoreactive cells and cortical thickness in the HI-ACSF and HI-NSP groups were comparable in a preterm HIE mouse model wherein P2 mice were subjected to an HI insult and administered neuroserpin (Supplementary Figure S4), suggesting that there is a critical period for the neuroprotective effect of neuroserpin on the HI-induced cortical abnormalities.

**Figure 7:**
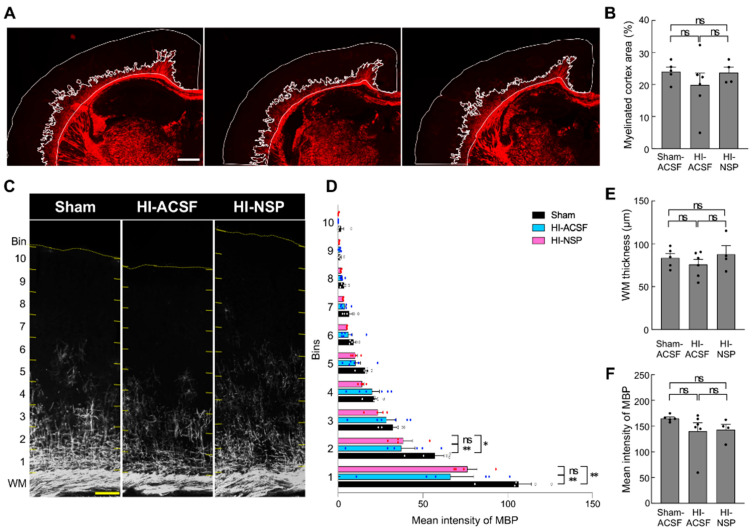
Effects of an intracerebroventricular injection of recombinant neuroserpin protein on HI-induced hypomyelination. (**A**) Representative images of MBP immunostaining in the brains of P12 sham mice, HI-ACSF mice and HI-NSP mice with mild HIE. C57BL/6J mice were subjected to HI procedures at P8, and then administered 100 ng of recombinant neuroserpin protein into the lateral ventricle of the brain 30 min after HI insults. The scale bars indicate 500 µm. (**B**) Myelinated cortex area in the iscortex was measured using ImageJ software in a blinded manner (sham mice: *n* = 5; HI-ACSF mice: *n* = 6; HI-NSP mice: *n* = 4; total of 15 animals used). Values represent the mean ± SEM. Statistical significance was evaluated using a one-way ANOVA followed by a Tukey post hoc test. ns: non-significant. (**C**,**D**) Representative images of MBP immunoreactivity in the S1 area. A cortical ROI area was equally divided into 10 bins from the white matter (WM) boundary to the cortical surface, and the mean intensity of MBP immunoreactivity was measured in each bin. The scale bar indicates 100 µm. Values represent the mean ± SEM. Statistical significance was evaluated using a two-way ANOVA followed by a Tukey post hoc test. * *p* < 0.05, ** *p* < 0.01. ns: non-significant. (sham mice: *n* = 5; HI-ACSF mice: *n* = 6; HI-NSP mice: *n* = 4; total of 15 animals used) (**E**,**F**) The thickness and mean intensity of MBP immunoreactivity in the WM were measured using ImageJ software in a blinded manner. (sham mice: *n* = 5; HI-ACSF mice: *n* = 6; HI-NSP mice: *n* = 4; total of 15 animals used).

We next examined whether mild HI at P8 affected myelination at P12 and whether neuroserpin had any effect on this process as well (Figure 7). The extent of cortical myelination was quantified following the method described by van Tilborg et al. [45]. The myelinated region in the isocortex was defined as the area extending from the white matter boundary to the most superficial MBP-immunoreactive boundary (Figure 7A,B). In addition, the mean intensity of MBP immunoreactivity in each bin within S1was measured (Figure 7C,D), as well as the thickness and mean intensity of MBP immunoreactivity in the white matter (Figure 7E,C). There were no significant differences in the proportion of the myelinated isocortex, or in the thickness and mean intensity of MBP immunoreactivity in the white matter among the sham, HI-ACSF, and HI-NSP groups (Figure 7A,B,E,F). In contrast, the mean intensity of MBP immunoreactivity in the deeper cortical layers (bins 1 and 2) was significantly decreased in both HI-ACSF and HI-NSP groups compared with the sham group, and the protective effects of neuroserpin on myelination was not observed. These findings suggest that neuroserpin exerts protective effects against oxidative stress and glial activation, and preserves neuronal distribution and cortical thickness, whereas no improvement in myelination was observed at least at P12.

## 4. Discussion

This study aimed to elucidate the spatiotemporal expression pattern of neuroserpin during postnatal brain development and to assess its protective effects, both endogenous and exogenous, in a neonatal HIE mouse model. Our findings reveal that neuroserpin expression in the cortex undergoes dynamic changes during the first two postnatal weeks. Notably, neuroserpin-immunoreactive cell density peaks in layers 5 and 6b at P8–P10 and gradually increases in layer 2/3, while remaining relatively stable in L4 and L6a. Contrary to initial expectations, neuroserpin deficiency did not exacerbate HI-induced brain damage. However, exogenous neuroserpin treatment significantly mitigated lipid peroxidation, microglial activation, reactive astrogliosis, cortical thinning, and neuronal distribution changes in mild HIE.

Emerging evidence suggests that even mild HIE can have long-term impacts. While children with mild HIE (Sarnat stage 1) have been historically assumed to experience minimal long-term effects [13,14], recent studies have highlighted subtle but significant deficits, including cognitive and neuropsychological challenges later in life [15,16]. Some untreated infants with mild HIE exhibit cognitive, language and communication deficits at 18–22 months [15], and children with mild HIE experience neuropsychological problems in school and peer relationships in adolescence [16]. Moreover, recent studies have reported electroencephalography (EEG) abnormalities in infants with even just mild HIE [46,47]. However, the pathophysiology of mild HIE remains poorly understood.

Our study showed a marked microglial phagocytic immunoreactivity, particularly in L4, along with accumulation of reactive astrocytes, a reduction in cortical thickness and an alteration in neuronal distribution in mild HIE mice that had been subjected to an HI insult at P8 (Figure 4, Figure 5 and Figure 6). In the rodent S1 barrel cortex, thalamocortical projections innervate the cortical plate at P2 and begin to form periphery-related patterns in the whisker barrel field [48,49]. At P8, the majority of L4 neurons are localised within the septa of the cytoarchitectonic barrels. Subsequently, sensory input-dependent L4 to L2/3 and intra-L2/3 horizontal connections are established at P10–P14 and P13–P16, respectively [49]. It can therefore be reasonably deduced that the HI-induced microglial inflammation and neuronal damage in L4 at P8 could result in the impairment of the corticocortical connections between L4 and L2/3. Our study also found that the treatment with neuroserpin markedly diminished the activated microglial immunoreactivity in the cortex of mild HIE mice (Figure 4). Notably, the number of neuroserpin-immunoreactive cells in L4 was relatively low compared to other layers, with no significant change from P4 to P14 (Figure 1). These findings suggest that the low level of neuroserpin expression in L4 is likely to be associated with its vulnerability following HI, and that a single administration of neuroserpin is sufficient to protect against the cortical damage in mild HIE. Although further functional studies are required to explore neuroserpin’s role in cortical circuit development, our data suggest that its administration could ameliorate HI-induced disruptions in connectivity during critical developmental windows.

In preterm mild/moderate HI models where rodents underwent the HI procedure at P2, previous studies have shown that HI causes the strongest cleaved caspase-3 immunoreactivity in L4 [50], and transient hypoconnectivity in L4 as well as persistent functional and anatomical changes in circuits to L4 until adolescence [51]. In contrast to the neonatal HIE model (Figure 6), the neuroprotective effect of neuroserpin was not found in the preterm HIE model (Supplementary Figure S4), suggesting that the neuroprotective effects of neuroserpin may depend on the timing and severity of HI injury. Additionally, in our neonatal HIE model (P8), single neuroserpin treatment was effective in mild cases but not sufficient to suppress brain damage in severe cases (Supplementary Figure S2). These findings point to the existence of a critical window for neuroserpin’s efficacy and the need for severity-based optimization of dosing.

Our results also diverge from findings in adult stroke models, where neuroserpin deficiency exacerbates outcomes and its supplementation reduces infarct size and neurological deficits [24,25,52,53]. Neuroserpin deficiency results in larger infarct size and worse neurological outcome after MCAO in adult mice [53]. However, our results in the neonatal HIE model did not reflect the aggravating effects of neuroserpin deficiency observed in the adult stroke model. This discrepancy may be attributed to a variety of factors, ranging from differences in brain maturity, alteration in the expression levels and distribution patterns of neuroserpin, tPA and other protective factors during brain maturation, to regional and layer-selective HI susceptibility between neonatal and adult brains.

Given the complexity of neonatal HIE pathology, which involve oxidative stress, inflammation, excitotoxicity, and impaired cortical maturation, therapeutic approaches targeting multiple mechanisms are highly desirable. Neuroserpin has demonstrated diverse protective effects, including inhibition of microglial activation, reduction of oxidative stress-induced cell death, and attenuation of NMDA-induced neurotoxicity in vitro [27,28,54,55]. The present study demonstrated that a single administration of neuroserpin significantly reduced lipid peroxidation and microglial activation as well as reactive astrogliosis in a neonatal mild HIE model, suggesting attenuation of oxidative stress and glial inflammation. Although no effect of neuroserpin on myelination was observed 4 days after HI insult, the suppression of oxidative stress and glial activation to sham levels suggests a potential secondary and delayed protective effect on myelination. Importantly, neuroserpin treatment protected against cortical thinning and alterations in neuronal distribution caused by HI insult. Moreover, its potential role in synaptic connectivity and plasticity suggests additional benefits for cognitive and behavioral outcomes [34,56,57]. Given that the L5 and L6b (a layer which is considered the remnant of subplate) neurons are the major sources of neuroserpin at P8–P10 (Figure 1) [22], secreted neuroserpin from L5 and L6b neurons may play a role in cortical maturation, including the development of thalamocortical and intracortical circuits [58]. While a recent study reports no significant impact of neuroserpin deficiency on cortical lamination or dendritic length during postnatal development [59], its involvement in cortical circuit formation warrants further investigation. Notably, neuroserpin KO mice exhibit cognitive and social behaviour deficits [56] despite the absence of overt structural abnormalities [59], suggesting that neuroserpin contributes to the functional maturation and stability of neural circuits rather than gross morphological development. These findings highlight neuroserpin’s potential as a versatile pharmacotherapeutic candidate for neonatal HIE. Given that therapeutic hypothermia is insufficient to fully prevent brain injury and that its efficacy for mild HIE remains uncertain, neuroserpin may offer complementary protection through distinct molecular mechanisms.

## 5. Conclusions

This study provides novel insights into the pathophysiology of mild neonatal HIE, demonstrating marked microglial activation (particularly in L4), accumulated reactive astrocytes, altered distribution of cortical neurons, and cortical thinning. Furthermore, we show that endogenous neuroserpin is insufficient for protecting against HI-induced brain damage in neonatal mice, but exogenous neuroserpin treatment at the time of HI significantly mitigates these pathological changes. Our findings suggest that neuroserpin could serve as a promising complementary therapy for neonatal HIE, offering neuroprotection across multiple pathological domains. Further research should focus on identifying the critical period and optimal dosing conditions to maximise neuroserpin’s therapeutic potential, including evaluation of intranasal (nose-to-brain) delivery for translational applications. In addition, long-term behavioural and anatomical assessments are needed to validate the functional implications of neurodevelopmental outcomes and to confirm whether the acute-phase neuroprotection conferred by neuroserpin treatment is sustained into later developmental and adult stages.

## Data Availability

The original contributions of this study are included in the article/Supplementary Material. Further inquiries can be directed to the corresponding authors.

## Supplementary Materials

The following supporting information can be downloaded at: https://www.mdpi.com/article/10.3390/cells14231840/s1, Figure S1: Increased neuroserpin immunoreactivity in cultured cortical neurons during maturation; Figure S2: No difference in activated microglial immunoreactivity between the control and NSP-treated mice with severe HIE; Figure S3: Distribution of S100β-positive cells and GFAP-positive cells; Figure S4: Neuroserpin treatment did not attenuate HI-induced alterations in distribution of cortical neurons and reduction in cortical thickness in a preterm HIE model; Table S1: Summary of animal numbers used in each experiment; Table S2: Statistical Results: P values in ANOVA and Kruskal–Wallis test.

## References

[1] Volpe J.J. (2001). Perinatal brain injury: From pathogenesis to neuroprotection. Ment. Retard. Dev. Disabil. Res. Rev..

[2] de Vries L.S., Jongmans M.J. (2010). Long-term outcome after neonatal hypoxic-ischaemic encephalopathy. Arch. Dis. Child. Fetal Neonatal Ed..

[3] Lee A.C., Kozuki N., Blencowe H., Vos T., Bahalim A., Darmstadt G.L., Niermeyer S., Ellis M., Robertson N.J., Cousens S. (2013). Intrapartum-related neonatal encephalopathy incidence and impairment at regional and global levels for 2010 with trends from 1990. Pediatr. Res..

[4] Sarnat H.B., Sarnat M.S. (1976). Neonatal encephalopathy following fetal distress. A clinical and electroencephalographic study. Arch. Neurol..

[5] Thompson C.M., Puterman A.S., Linley L.L., Hann F.M., van der Elst C.W., Molteno C.D., Malan A.F. (1997). The value of a scoring system for hypoxic ischaemic encephalopathy in predicting neurodevelopmental outcome. Acta Paediatr..

[6] Boutilier R.G. (2001). Mechanisms of cell survival in hypoxia and hypothermia. J. Exp. Biol..

[7] Azzopardi D., Strohm B., Marlow N., Brocklehurst P., Deierl A., Eddama O., Goodwin J., Halliday H.L., Juszczak E., Kapellou O. (2014). Effects of hypothermia for perinatal asphyxia on childhood outcomes. N. Engl. J. Med..

[8] Azzopardi D.V., Strohm B., Edwards A.D., Dyet L., Halliday H.L., Juszczak E., Kapellou O., Levene M., Marlow N., Porter E. (2009). Moderate hypothermia to treat perinatal asphyxial encephalopathy. N. Engl. J. Med..

[9] Millar L.J., Shi L., Hoerder-Suabedissen A., Molnár Z. (2017). Neonatal hypoxia ischaemia: Mechanisms, models, and uherapeutic challenges. Front. Cell. Neurosci..

[10] Ranjan A.K., Gulati A. (2023). Advances in therapies to treat neonatal hypoxic-ischemic encephalopathy. J. Clin. Med..

[11] Davidson J.O., Wassink G., van den Heuij L.G., Bennet L., Gunn A.J. (2015). Therapeutic hypothermia for neonatal hypoxic-ischemic encephalopathy - where to from here?. Front. Neurol..

[12] Thayyil S., Pant S., Montaldo P., Shukla D., Oliveira V., Ivain P., Bassett P., Swamy R., Mendoza J., Moreno-Morales M. (2021). Hypothermia for moderate or severe neonatal encephalopathy in low-income and middle-income countries (HELIX): A randomised controlled trial in India, Sri Lanka, and Bangladesh. Lancet Glob. Health.

[13] Robertson C., Finer N. (1985). Term infants with hypoxic-ischemic encephalopathy: Outcome at 3.5 years. Dev. Med. Child Neurol..

[14] Robertson C.M., Finer N.N., Grace M.G. (1989). School performance of survivors of neonatal encephalopathy associated with birth asphyxia at term. J. Pediatr..

[15] Chalak L.F., Nguyen K.A., Prempunpong C., Heyne R., Thayyil S., Shankaran S., Laptook A.R., Rollins N., Pappas A., Koclas L. (2018). Prospective research in infants with mild encephalopathy identified in the first six hours of life: Neurodevelopmental outcomes at 18–22 months. Pediatr. Res..

[16] Halpin S., McCusker C., Fogarty L., White J., Cavalière E., Boylan G., Murray D. (2022). Long-term neuropsychological and behavioral outcome of mild and moderate hypoxic ischemic encephalopathy. Early Hum. Dev..

[17] Yang M., Wang K., Liu B., Shen Y., Liu G. (2024). Hypoxic-ischemic encephalopathy: Pathogenesis and promising therapies. Mol. Neurobiol..

[18] Disdier C., Stonestreet B.S. (2020). Hypoxic-ischemic-related cerebrovascular changes and potential therapeutic strategies in the neonatal brain. J. Neurosci. Res..

[19] Semple B.D., Blomgren K., Gimlin K., Ferriero D.M., Noble-Haeusslein L.J. (2013). Brain development in rodents and humans: Identifying benchmarks of maturation and vulnerability to injury across species. Prog. Neurobiol..

[20] Osterwalder T., Contartese J., Stoeckli E.T., Kuhn T.B., Sonderegger P. (1996). Neuroserpin, an axonally secreted serine protease inhibitor. EMBO J..

[21] Kumar A., Ragg H. (2008). Ancestry and evolution of a secretory pathway serpin. BMC Evol. Biol..

[22] Kondo S., Al-Hasani H., Hoerder-Suabedissen A., Wang W.Z., Molnár Z. (2015). Secretory function in subplate neurons during cortical development. Front. Neurosci..

[23] Adorjan I., Tyler T., Bhaduri A., Demharter S., Finszter C.K., Bako M., Sebok O.M., Nowakowski T.J., Khodosevich K., Møllgård K. (2019). Neuroserpin expression during human brain development and in adult brain revealed by immunohistochemistry and single cell RNA sequencing. J. Anat..

[24] Yepes M., Sandkvist M., Wong M.K., Coleman T.A., Smith E., Cohan S.L., Lawrence D.A. (2000). Neuroserpin reduces cerebral infarct volume and protects neurons from ischemia-induced apoptosis. Blood.

[25] Cinelli P., Madani R., Tsuzuki N., Vallet P., Arras M., Zhao C.N., Osterwalder T., Rülicke T., Sonderegger P. (2001). Neuroserpin, a neuroprotective factor in focal ischemic stroke. Mol. Cell. Neurosci..

[26] Liao Y., Zhang Q., Shi Q., Liu P., Zhong P., Guo L., Huang Z., Peng Y., Liu W., Zhang S. (2026). Neuroserpin alleviates cerebral ischemia-reperfusion injury by suppressing ischemia-induced endoplasmic reticulum stress. Neural Regen. Res..

[27] Ma J., Yu D., Tong Y., Mao M. (2012). Effect of neuroserpin in a neonatal hypoxic-ischemic injury model ex vivo. Biol. Res..

[28] Wang L., Zhang Y., Asakawa T., Li W., Han S., Li Q., Xiao B., Namba H., Lu C., Dong Q. (2015). Neuroprotective effect of neuroserpin in oxygen-glucose deprivation- and reoxygenation-treated rat astrocytes in vitro. PLoS ONE.

[29] Yang X., Asakawa T., Han S., Liu L., Li W., Wu W., Luo Y., Cao W., Cheng X., Xiao B. (2016). Neuroserpin protects rat neurons and microglia-mediated inflammatory response sgainst oxygen-glucose deprivation- and reoxygenation treatments in an in vitro study. Cell. Physiol. Biochem..

[30] Hastings G.A., Coleman T.A., Haudenschild C.C., Stefansson S., Smith E.P., Barthlow R., Cherry S., Sandkvist M., Lawrence D.A. (1997). Neuroserpin, a brain-associated inhibitor of tissue plasminogen activator is localized primarily in neurons. Implications for the regulation of motor learning and neuronal survival. J. Biol. Chem..

[31] Dupré N., Arabo A., Orset C., Maucotel J., Detroussel Y., Hauchecorne M., Gonzalez B.J., Marret S., Vivien D., Leroux P. (2020). Neonatal cerebral hypoxia-ischemia in mice triggers age-dependent vascular effects and disabilities in adults; implication of tissue plasminogen activator (tPA). Exp. Neurol..

[32] Kilicdag H., Akillioglu K., Kilic Bagır E., Kose S., Erdogan S. (2024). Neuroserpinas an adjuvant therapy for hypothermia on brain injury in neonatal hypoxic-ischemic rats. Am. J. Perinatol..

[33] Rice J.E., Vannucci R.C., Brierley J.B. (1981). The influence of immaturity on hypoxic-ischemic brain damage in the rat. Ann. Neurol..

[34] Madani R., Kozlov S., Akhmedov A., Cinelli P., Kinter J., Lipp H.P., Sonderegger P., Wolfer D.P. (2003). Impaired explorative behavior and neophobia in genetically modified mice lacking or overexpressing the extracellular serine protease inhibitor neuroserpin. Mol. Cell. Neurosci..

[35] Edwards A.B., Feindel K.W., Cross J.L., Anderton R.S., Clark V.W., Knuckey N.W., Meloni B.P. (2017). Modification to the Rice-Vannucci perinatal hypoxic-ischaemic encephalopathy model in the P7 rat improves the reliability of cerebral infarct development after 48 hours. J. Neurosci. Methods.

[36] Kim J.Y., Grunke S.D., Levites Y., Golde T.E., Jankowsky J.L. (2014). Intracerebroventricular viral injection of the neonatal mouse brain for persistent and widespread neuronal transduction. J. Vis. Exp..

[37] Liao Y., Wang J.Y., Pan Y., Zou X., Wang C., Peng Y., Ao Y.L., Lam M.F., Zhang X., Zhang X.Q. (2023). The protective effect of (-)-Tetrahydroalstonine against OGD/R-induced neuronal injury via autophagy regulation. Molecules.

[38] Liang W., Chuan-Zhen L., Qiang D., Jian Q., Hui-Min R., Bao-Guo X. (2004). Reductions in mRNA of the neuroprotective agent, neuroserpin, after cerebral ischemia/reperfusion in diabetic rats. Brain Res..

[39] Ishihara K., Amano K., Takaki E., Ebrahim A.S., Shimohata A., Shibazaki N., Inoue I., Takaki M., Ueda Y., Sago H. (2009). Increased lipid peroxidation in Down’s syndrome mouse models. J. Neurochem..

[40] McRae A., Gilland E., Bona E., Hagberg H. (1995). Microglia activation after neonatal hypoxic-ischemia. Brain Res. Dev. Brain Res..

[41] Brégère C., Schwendele B., Radanovic B., Guzman R. (2022). Microglia and stem-cell mediated neuroprotection after neonatal hypoxia-ischemia. Stem Cell Rev. Rep..

[42] Gonda Y., Andrews W.D., Tabata H., Namba T., Parnavelas J.G., Nakajima K., Kohsaka S., Hanashima C., Uchino S. (2013). Robo1 regulates the migration and laminar distribution of upper-layer pyramidal neurons of the cerebral cortex. Cereb. Cortex.

[43] Hoerder-Suabedissen A., Molnár Z. (2015). Development, evolution and pathology of neocortical subplate neurons. Nat. Rev. Neurosci..

[44] Yoshinaga S., Shin M., Kitazawa A., Ishii K., Tanuma M., Kasai A., Hashimoto H., Kubo K.I., Nakajima K. (2021). Comprehensive characterization of migration profiles of murine cerebral cortical neurons during development using FlashTag labeling. iScience.

[45] van Tilborg E., van Kammen C.M., de Theije C.G.M., van Meer M.P.A., Dijkhuizen R.M., Nijboer C.H. (2017). A quantitative method for microstructural analysis of myelinated axons in the injured rodent brain. Sci. Rep..

[46] Garvey A.A., Pavel A.M., O’Toole J.M., Walsh B.H., Korotchikova I., Livingstone V., Dempsey E.M., Murray D.M., Boylan G.B. (2021). Multichannel EEG abnormalities during the first 6 hours in infants with mild hypoxic-ischaemic encephalopathy. Pediatr. Res..

[47] Natarajan N., Benedetti G., Perez F.A., Wood T.R., German K.R., Lockrow J.P., Puia-Dumitrescu M., Myers E., Mietzsch U. (2022). Association between early EEG background and outcomes in infants with mild HIE undergoing therapeutic hypothermia. Pediatr. Neurol..

[48] Molnár Z., Kwan K.Y. (2024). Development and evolution of thalamocortical connectivity. Cold Spring Harb. Perspect. Biol..

[49] Erzurumlu R.S., Gaspar P. (2012). Development and critical period plasticity of the barrel cortex. Eur. J. Neurosci..

[50] Okusa C., Oeschger F., Ginet V., Wang W.Z., Hoerder-Suabedissen A., Matsuyama T., Truttmann A.C., Molnár Z. (2014). Subplate in a rat model of preterm hypoxia-ischemia. Ann. Clin. Transl. Neurol..

[51] Sheikh A., Meng X., Kao J.P.Y., Kanold P.O. (2022). Neonatal hypoxia-ischemia causes persistent intracortical circuit changes in layer 4 of rat auditory cortex. Cereb. Cortex.

[52] Zhang Z., Zhang L., Yepes M., Jiang Q., Li Q., Arniego P., Coleman T.A., Lawrence D.A., Chopp M. (2002). Adjuvant treatment with neuroserpin increases the therapeutic window for tissue-type plasminogen activator administration in a rat model of embolic stroke. Circulation.

[53] Gelderblom M., Neumann M., Ludewig P., Bernreuther C., Krasemann S., Arunachalam P., Gerloff C., Glatzel M., Magnus T. (2013). Deficiency in serine protease inhibitor neuroserpin exacerbates ischemic brain injury by increased postischemic inflammation. PLoS ONE.

[54] Lebeurrier N., Liot G., Lopez-Atalaya J.P., Orset C., Fernandez-Monreal M., Sonderegger P., Ali C., Vivien D. (2005). The brain-specific tissue-type plasminogen activator inhibitor, neuroserpin, protects neurons against excitotoxicity both in vitro and in vivo. Mol. Cell. Neurosci..

[55] Cheng Y., Loh Y.P., Birch N.P. (2017). Neuroserpin attenuates H_2_O_2_-induced oxidative stress in hippocampal neurons via AKT and BCL-2 signaling pathways. J. Mol. Neurosci..

[56] Reumann R., Vierk R., Zhou L., Gries F., Kraus V., Mienert J., Romswinkel E., Morellini F., Ferrer I., Nicolini C. (2017). The serine protease inhibitor neuroserpin is required for normal synaptic plasticity and regulates learning and social behavior. Learn. Mem..

[57] D’Acunto E., Fra A., Visentin C., Manno M., Ricagno S., Galliciotti G., Miranda E. (2021). Neuroserpin: Structure, function, physiology and pathology. Cell. Mol. Life Sci..

[58] Molnár Z., Luhmann H.J., Kanold P.O. (2020). Transient cortical circuits match spontaneous and sensory-driven activity during development. Science.

[59] Kement D., Reumann R., Schostak K., Voß H., Douceau S., Dottermusch M., Schweizer M., Schlüter H., Vivien D., Glatzel M. (2021). Neuroserpin is strongly expressed in the developing and adult mouse neocortex but its absence does not perturb cortical lamination and synaptic proteome. Front. Neuroanat..

