# Supplementary material for: Neuroserpin: A Potential Neuroprotective Agent in Mild Neonatal Hypoxic–Ischaemic Encephalopathy
Source: Cells. 2025 Nov 21;14(23):1840. doi: 10.3390/cells14231840 (PMC12691123; doi:10.3390/cells14231840)
Supplement: Supplementary file 1 [file cells-14-01840-s001.zip › cells-3967478-supplementary.pdf]

SUPPLEMENTARY MATERIALS

Supplementary Figures

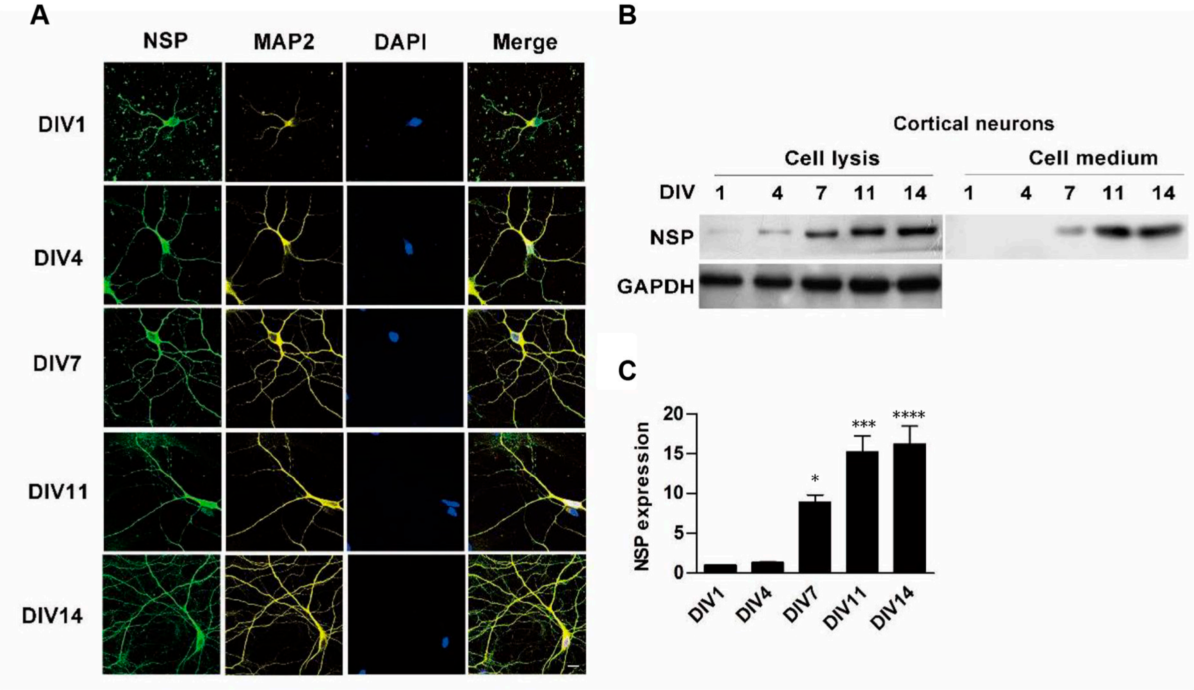

Supplementary Figure S1. Increased neuroserpin immunoreactivity in cultured cortical neurons during maturation

Primary rat cortical neurons were cultured as described in the Materials and Methods section, and used for immunostaining and immunoblotting after the indicated days *in vitro* (DIV). (A) Representative images of the cortical neurons cultured for the indicated period and immunocytostained using primary antibodies for neuroserpin (1:500; sc-48360, Santa Cruz Biotechnology) and microtubule-associated protein 2 (MAP2) (1:1000; AB5622, Millipore) and the secondary antibodies: Alexa Fluor 488-conjugated anti-mouse IgG (1:2000; A11029, Invitrogen) and Alexa Fluor 568-conjugated anti-Rabbit IgG (1:2000; A11036, Invitrogen). The scale bar indicates 10 µm. (B) Representative immunoblotting images of neuroserpin and GAPDH expression in cell lysate and cell medium indicate increase in neuroserpin expression (n=3). Immunoblotting was performed as described in the Materials and Methods section. (C) The band intensity was measured using the Quantity One software and normalised to that of GAPDH. Values represent the mean ± SEM. Statistical significance was evaluated using a one-way ANOVA followed by a Dunnett's multiple comparisons test. *$P < 0.05$, ***$P < 0.001$, ****$P < 0.0001$. ns: non-significant.

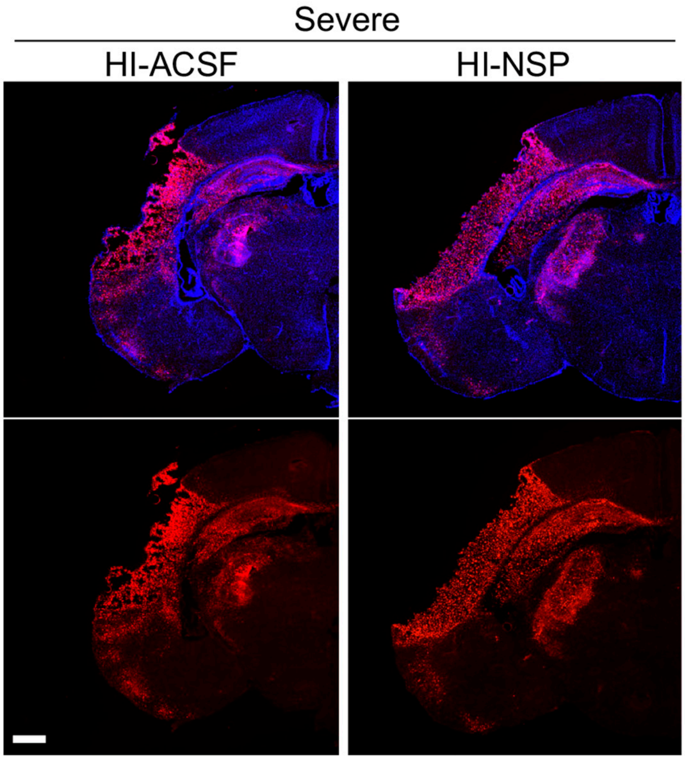

Supplementary Figure S2. No difference in activated microglial immunoreactivity between the control and NSP-treated mice with severe HIE

Representative CD68 immunostaining images from P12 HI-ACSF mice and HI-NSP mice classified as having severe cortical injury are shown. C57BL/6J mice at P8 were subjected to left CCA and ECA ligation, allowed to rest for at least 1h, and then exposed to hypoxic gas (10% oxygen in nitrogen) for 40 min. Recombinant neuroserpin protein (100 ng) or ACSF was administered intracerebroventricularly 30 min after the HI procedures. Brains were collected at P12. This modified Vannucci method tended to produce mild HIE, although occasional severe injury was observed. The severity of HI brain damage was categorised as mild or severe based on gross cortical shape: brains exhibiting an obvious cortical depression were classified as severe, whereas those without were classified as mild. Upper panels: merged CD68 and DAPI images. Lower panels: CD68 immunostaining alone. HI-ACSF: mice that underwent HI insult and ACSF injection; HI-NSP: mice that underwent HI insult and neuroserpin injection. The scale bar indicates 500 μm.

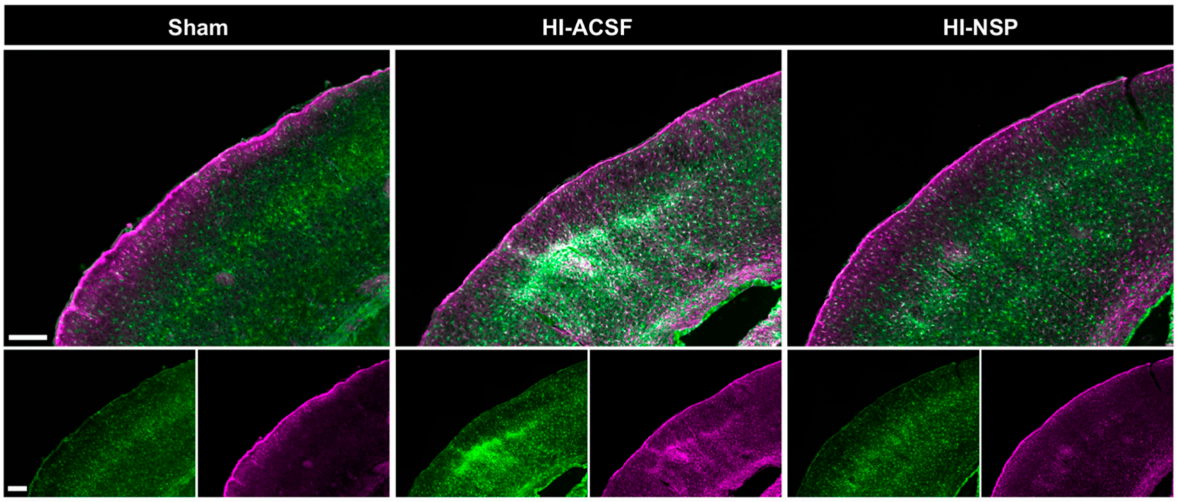

Supplementary Figure S3. Distribution of S100β-positive cells and GFAP-positive cells

Representative images of S100β (green) and GFAP (magenta) immunostaining in the brain sections from P12 sham mice, HI-ACSF mice and HI-NSP mice. HI-ACSF: mice that underwent HI insult and ACSF injection; HI-NSP: mice that underwent HI insult and neuroserpin injection. The scale bars indicate 200 μm.

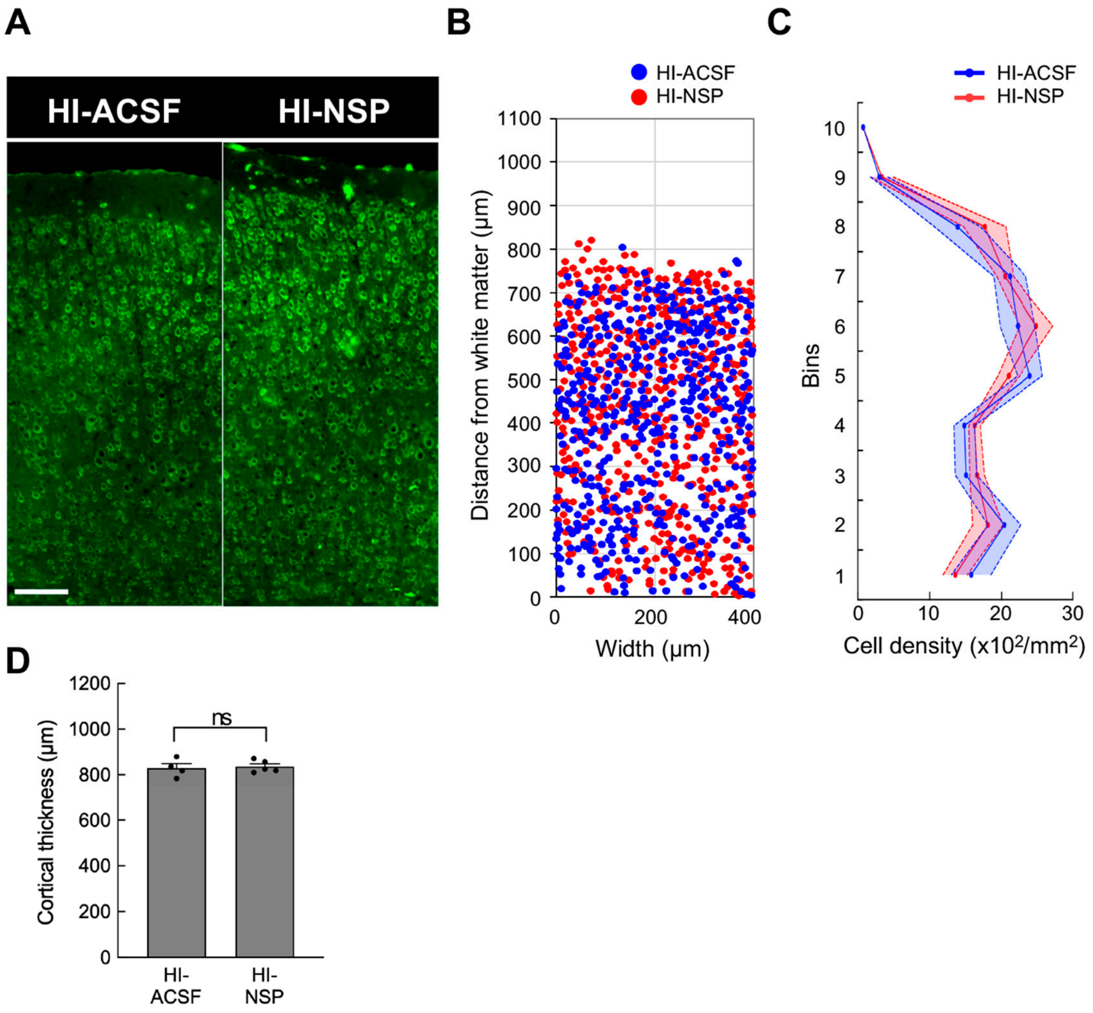

Supplementary Figure S4. Neuroserpin treatment did not attenuate HI-induced alterations in distribution of cortical neurons and reduction in cortical thickness in a preterm HIE model

(A) Representative images of NeuN immunostaining in the brain sections of P12 HI-ACSF mice and HI-NSP mice that had been subjected to HI insult at P2. C57BL/6J mice were subjected to left CCA and ECA ligation at P2, allowed to rest for at least 1 h, and then exposed to hypoxic gas (10% oxygen in nitrogen) for 40 min. The pups were then injected with 50 ng of neuroserpin or ACSF in a total volume of 1 μL into the lateral ventricle (0.9 mm laterally and 1.55 mm caudally from the lambda and -1.2 mm vertically from the cranial surface), 30 min following the HI procedure. HI-ACSF: mice that underwent HI insult and ACSF injection; HI-NSP: mice that underwent HI insult and neuroserpin injection. The scale bar indicates 100 μm. (B) Illustration of the distribution of NeuN-immunoreactive cells. (C) The density distribution of NeuN-immunoreactive cells in the area shown in Supplementary Figure 3A (HI-ACSF mice: n=4; HI-NSP mice: n=3; total of 7 animals used). Values represent the mean ± SEM. (D) Cortical thickness of the somatosensory cortex. Cortical thickness was measured at three different locations in the rectangular area using ImageJ software in a blind manner, and mean value was calculated (HI-ACSF mice: n=4; HI-NSP mice: n=5; total of 9 animals used). Values represent the mean ± SEM. Statistical significance was evaluated using Student's t-test.

Supplementary Table S1

Summary of animal numbers used in each experiment.

Figures	Age (HI procedure → tissue collection)	Number of animals each group (n)	Total number of animals (n)
Figure 1H, I	P4/ P8/ P10/ P14 (Naïve)	P4: n=4 ; P8: n=4; P10: n=4; P14: n=4	n=16
Figure 2C	P8 (CCA+ECA ligation) → P9	sham: n=3; HI: n=6	n=9
Figure 2D	P8 (CCA+ECA ligation) → P12	sham: n=4; HI: n=6	n=10
Figure 3B	P8 (CCA ligation) → P10	WT: n=14; KO: n=29	n=43
Figure 3C	P8 (CCA ligation) → P10	WT: n=10; KO: n=15	n=25
Figure 3D	P8 (CCA ligation) → P10	WT: n=10; KO: n=15	n=25
Figure 4C	P8 (CCA+ECA ligation) → P12	Naïve: n=5; HI-ACSF: n=6; HI-NSP: n=4	n=15
Figure 4F	P8 (CCA+ECA ligation) → P12	Sham: n=4; HI-ACSF: n=6; HI-NSP: n=4	n=14
Figure 5B	P8 (CCA+ECA ligation) → P12	Sham: n=5; HI-ACSF: n=6; HI-NSP: n=4	n=15
Figure 6D, E	P8 (CCA+ECA ligation) → P12	Sham: n=4; HI-ACSF: n=6; HI-NSP: n=4	n=14
Figure 7B, D-F	P8 (CCA+ECA ligation) → P12	Sham: n=5; HI-ACSF: n=6; HI-NSP: n=4	n=15
Supplementary Figure 4D	P2 (CCA+ECA ligation) → P12	HI-ACSF: n=4; HI-NSP: n=5	n=9

Supplementary Table S2

Statistical Results: P values in ANOVA and Kruskal–Wallis test

	Figure 1(H)		Figure 1(I)	
Test	Two-way ANOVA		Two-way ANOVA	
	F (Dfn, Dfd) = F value	P value	F (Dfn, Dfd) = F value	P value
Age	F (3, 60) = 19.14	P<0.0001	F (3, 60) = 0.000	P>0.9999
Layer	F (4, 60) = 82.40	P<0.0001	F (4, 60) = 156.5	P<0.0001
Age x Layer	F (12, 60) = 5.931	P<0.0001	F (12, 60) = 16.69	P<0.0001

	Figure 2(B)		Figure 2(C) 46 kDa		Figure 2(C) 55 kDa	
Test	One-way ANOVA		One-way ANOVA		One-way ANOVA	
	F (Dfn, Dfd) = F value	P value	F (Dfn, Dfd) = F value	P value	F (Dfn, Dfd) = F value	P value
	F (10, 33) = 5.336	P=0.0001	F (3, 14) = 0.5097	P=0.6820	F (3, 14) = 0.1532	P=0.9259

	Figure 2(D)	
Test	Two-way ANOVA	
	F (Dfn, Dfd) = F value	P value
HI	F (1, 40) = 4.013	P=0.0520
Layer	F (4, 40) = 26.77	P<0.0001
Age x Layer	F (4, 40) = 0.5703	P=0.6857

	Figure 4(C) 26 kDa		Figure 4(C) 50 kDa		Figure 4(F) Hemisphere	
Test	One-way ANOVA		One-way ANOVA		Kruskal–Wallis test	
	F (Dfn, Dfd) = F value	P value	F (Dfn, Dfd) = F value	P value	H (Df) = K-W statistic	P value
	F (4, 20) = 8.597	P=0.0003	F (4, 20) = 6.502	P=0.0016	H (2) = 9.929	P=0.0005

	Figure 4(F) Cortex		Figure 4(F) Thalamus		Figure 4(F) Hippocampus	
Test	Kruskal–Wallis test		Kruskal–Wallis test		Kruskal–Wallis test	
	H (Df) = K-W statistic	P value	H (Df) = K-W statistic	P value	H (Df) = K-W statistic	P value
	H (2) = 9.650	P=0.0010	H (2) = 10.06	P=0.0004	H (2) = 8.877	P=0.0028

	Figure 5		Figure 6(E) Anterior		Figure 6(E) Posterior	
Test	One-way ANOVA		One-way ANOVA		One-way ANOVA	
	F (Dfn, Dfd) = F value	P value	F (Dfn, Dfd) = F value	P value	F (Dfn, Dfd) = F value	P value
	F (2, 12) = 9.759	P=0.0030	F (2, 11) = 7.920	P=0.0074	F (2, 11) = 4.162	P=0.0451

	Figure 7(B)		Figure 7(E)		Figure 7(F)	
Test	One-way ANOVA		One-way ANOVA		Kruskal–Wallis test	
	F (Dfn, Dfd) = F value	P value	F (Dfn, Dfd) = F value	P value	H (Df) = K-W statistic	P value
	F (2, 12) = 0.7244	P=0.5046	F (2, 12) = 0.7860	P=0.4778	H (2) = 5.161	P=0.0682

	Figure 7(D)	
Test	Two-way ANOVA	
	F (Dfn, Dfd) = F value	P value
Bin	F (9, 120) = 95.29	P<0.0001
HI	F (2, 120) = 10.38	P<0.0001
Bin x HI	F (18, 120) = 2.216	P=0.0058

	Suppl. Figure 1(C)	
Test	One-way ANOVA	
	F (Dfn, Dfd) = F value	P value
	F (4, 10) = 25.91	P<0.0001
